# An Analysis of Auditory Manifestations in a Group of Adults with AIDS Prior to Antiretroviral Therapy

**Published:** 2011

**Authors:** Katijah Khoza-Shangase

**Keywords:** auditory function, otologic disease, prevalence, sensorineural, South Africa

## Abstract

The chief objective of the current study was to investigate the auditory status in a group of adults with AIDS before commencing antiretroviral therapy (ART) in a hospital outpatient clinic in Gauteng, South Africa. A total sample of 150 participants, aged between 20 and 46 years, was assessed following a prospective qualitative research design. All participants underwent case history interviews and medical record reviews, otoscopy and tympanometry, as well as conventional pure tone audiometry testing. Descriptive statistics was used to analyse data obtained. Prevalence, type, degree, configuration, and symmetry of the auditory manifestations; as well as type of onset of hearing loss and the possible causes of the auditory manifestations were analysed. Findings indicated that auditory manifestations in adults with AIDS are varied in nature and are possibly due to a number of causes. Manifestations including hearing loss, tinnitus and vertigo (in varied combinations) were found; with the types of hearing loss being mainly sensorineural in nature. The severity of hearing loss ranged from mild to severe, occurred either unilaterally or bilaterally; with the type of onset being mainly gradual and progressive in nature. The varied causes of hearing loss included HIV infection or AIDS illness as a primary cause, opportunistic infections, and various ototoxic therapies that the patients had undergone. Implications for future research as well as future assessment and management of patients with AIDS are raised.

## Introduction

The HIV pandemic in South Africa, now in its third decade, remains one of the biggest challenges this country has to confront. This occurs together with poverty, joblessness and other socio-economic challenges that the departments of health and social development strive to eradicate (Posel, Kahn and Walker, 2007). The HIV pandemic appears to have debatably generated more challenges to science and medicine than any other single disease. The virus has taken a huge toll in human suffering and has had an astounding socio-economic impact on healthcare in South Africa, and throughout the world, with conceivably more significant challenges occurring in third-world countries than in developed countries (Goudge, Gilson, Russel, Gumede and Mills, 2009). It is also believed that the incidence of HIV has also crafted a devastating burden and inimitable challenge to audiological health-service delivery in South Africa (Swanepoel, 2006; Khoza-Shangase, Mupawose, and Mlangeni. 2009). This challenge can partially be surmounted by ensuring that relevant amount of research is conducted to establish the physiological effects of HIV/ AIDS and its drug treatments on those who are infected.

Globally, research has shown numerous associations between HIV/AIDS and auditory performance in adults, including sensorineural hearing loss possibly linked with antiretroviral therapy (ART). However, the acquired evidence is predominantly from the developed world and research from developing countries remains limited. The known effects of HIV/AIDS on the auditory system are based mainly on cross-sectional studies and case reports conducted internationally in industrialised countries, with very scant information emanating from third-world countries where the presentation of the virus and its treatments may be different. Although much research has been conducted internationally, replication of such studies in the developing world is still required. The current study investigates specifically the auditory manifestations of AIDS in adults.

There are suggestions that hearing changes may be one of the presentations at any stage of HIV disease (Birchall, Wight, French, Cockbain and Smith, 1992; Noffsinger and Friedman, 1996; Campanini, Marani, Mastroianni, Cancellieri and Vicini, 2005; Khoza-Shangase, 2010). The documented prevalence and incidence of hearing loss in cases of HIV/ AIDS varies extensively, but this discrepancy may be credited to the vastly diverse samples, variable research methodologies, as well as different participant inclusion criteria used in the published studies. In general, the incidence of otologic manifestations of HIV/AIDS has been described to be reasonably small (Abemayor and Calcaterra, 1983; Kohan, Rothstein and Cohen, 1988; Lalwani and Sooy, 1992; Gold and Tami, 1998; Chandrasekhar, Connelly, Brahmbhatt, Chetan, Shah, Kloser and Baredes, 2000; Khoza and Ross, 2002). However, Flower (1991) reported on the high prevalence of hearing loss in patients with HIV/ AIDS when he declared that about 75% of adults with AIDS illness and 50% of persons with AIDS-related complex will present with clinical auditory-system abnormalities. Other researchers, such as Sooy (1987), have indicated that hearing impairment occurs in up to half of patients with AIDS. Khoza and Ross (2002) reported findings from South Africa, where hearing loss was found in 23% of their study sample of persons with HIV/ AIDS. The different results reported by these studies could well be due to methodological differences as opposed to differences in actual incidence of hearing loss (Khoza-Shangase, 2010). The most obvious difference among these studies is that while some focused on general HIV/ AIDS (with samples that included patients in all stages of HIV disease); others were more specific in their sampling.

Consistent with the reports that state minor auditory manifestations of HIV/ AIDS, are claims that this small incidence rate is exclusive to adults, but may be vastly different in children. These studies have resolved that there is an extremely low incidence (or no incidence) of otologic disease in adults with HIV infection, with possibly much higher incidence in paediatric patients (Khoza-Shangase, 2010). The high occurrence of conductive hearing loss in paediatric patients, globally, has been ascribed in part to the high incidence of serous otitis media, which may occur in up to 80% of cases (Smith and Canalis, 1989). Rosenberg, Schneider and Cohen (1985) examined medical records of adults with AIDS and discovered that while 71% of the patients had symptoms localised in the head and neck, none had otologic signs and symptoms. Similar findings were reported by Marcusen and Sooy (1985) who found no otologic abnormalities in their sample. Lalwani and Sooy (1992) maintain that otologic manifestations associated with HIV/ AIDS do occur but are less prevalent than other head and neck conditions.

With regard to comparing the occurrence of auditory manifestations to other head and neck effects of HIV/AIDS, there is significant variation in the reports. For instance, Rosenberg et al. (1985) reported a total lack of otologic symptoms in patients who presented with head and neck signs and symptoms, while Booth (1997) mentioned an upsurge in the number of people living with HIV/ AIDS who presented with otologic symptoms. Lalwani and Sooy (1992) maintained that ear-related manifestations of HIV/ AIDS occur to a lesser degree when compared to other head and neck conditions. It should be noted that none of these studies utilized an audiological test battery method which would facilitate definitive conclusions about the auditory symptoms. Chandrasekhar et al. (2000) claimed that as many as one-third of patients with HIV may have significant otologic complaints or difficulties, and they maintained that this is a significant percentage that may be higher than figures reported in the literature on otolaryngologic and infectious diseases. Research that has incorporated audiological assessments, such as Sooy's (1987) research, illustrate abnormal audiologic findings greater than 25 dB HL on pure-tone testing in 49% of the total sample tested, while Birchall et al. (1992) stated a regular presence of hearing loss on pure-tone testing in 39% of a small sample of patients. Not so long ago, Prasad, Bhojwani, Shenoy and Prasad (2006) reported that in their study, although 80% of HIV infections presented with otolaryngologic symptoms, otologic manifestations were seen in only 20% of the cases seen, with 13% of these symptoms linked to chronic suppurative otitis media.

A review of the literature on auditory findings implies variability as far as nature, degree, and configuration of the hearing losses diagnosed in patients with HIV/ AIDS. The documented hearing impairment may be conductive, sensorineural, or central; the severity of the loss may range from mild to severe; the onset of the loss may be sudden or gradual; and the hearing loss may be stable or may fluctuate (Timon and Walsh, 1989; Lalwani and Sooy, 1992; Bankaitis; 1996; Friedman and Noffsinger, 1998; Chandrasekhar et al., 2000; Khoza and Ross, 2002; Khoza-Shangase, 2010). Khoza and Ross (2002) also argue that the hearing loss may also be profound in nature.

Friedman and Noffsinger (1998), Gold and Tami (1998) and Lalwani and Sooy (1992) have all stated that the most common otologic problems reported in this population are serous otitis media and recurrent acute otitis media, which are primarily due to eustachian tube dysfunction; suggesting that one would anticipate the conductive type of hearing loss to be more common (Khoza-Shangase, 2010). Nevertheless, that finding is not substantiated in the literature. The rate of occurrence of sensorineural hearing loss (SNHL) is reported by Gold and Tami (1998) to range between 20% and 50%, based on reviews of studies by Bankaitis and Keith (1995), Lalwani and Sooy (1992) and Tami and Lee (1994). Similarly, Khoza and Ross (2002) found higher prevalence of SNHL in a sample of adults with HIV infection, where a definite increase in the number of occurrences of SNHL from stage 1 (asymptomatic HIV infection) to stage 3 (clinical AIDS) was also established in adults who were not on ART.

Very limited published literature exists with regards the degree of hearing loss in patients with HIV/AIDS. Sooy (1987) reported abnormal audiologic results of thresholds worse than 25 dB HL, with a sloping configuration on pure-tone audiometry of between 30 and 50 dB HL at 8000 Hz. A third of the patients in Sooy's study had moderate to severe hearing loss at three or more test frequencies, in at least one ear. Chandrasekhar et al. (2000), however indicated that all audiometric data in their study (i.e. pure tone, speech discrimination, and otoacoustic emissions), which was collapsed across CDC group, otologic complaint and age, there was no statistically significant effect on the ear. However, when data for right and left ear were combined, a statistically significant effect for the high frequencies (4000–8000 Hz) was found; with these high frequencies being significantly elevated relative to lower frequencies. Likewise, previously reported results have revealed that the audiometric data trends suggest deteriorating hearing loss in high frequencies. For example, Marcusen and Sooy (1985) found that some of their patients presented with a hearing loss on pure-tone testing involving 8000 Hz.

Chandrasekhar et al. (2000), Gold and Tami (1998) and Lalwani and Sooy (1992) state that such hearing loss steadily worsens with an increase in frequency, with high frequencies at a moderate degree of severity. Mata, Yebra Bango, Tutor de Ureta, Villarreal and Garcia (2000) reported that high-frequency sensorineural hearing loss was a common finding among the 30 patients studied. However, Khoza and Ross (2002) claim that the configuration of the hearing loss in HIV disease may not be frequency-range-specific, but may involve all frequencies. Again, these variations in reports may be attributed to the differences in the samples studied.

The description of hearing impairment in HIV/ AIDS varies even when one considers type of onset and symmetry of hearing loss (Khoza-Shangase, 2010). Several studies have reported on sudden SNHL in patients with HIV or AIDS (Real, Thomas and Gerwins, 1987; Timon and Walsh, 1989; Solanellas, Soldado and Lozano, 1996; Khoza and Ross, 2002). Smith and Canalis (1989) found that the otologic symptoms can include unilateral or bilateral SNHL, which can occasionally be of a sudden onset in nature, but that this loss is commonly rapidly progressive in nature. Khoza and Ross (2002) found the sudden onset of the hearing loss to be more prevalent in participants with more severe SNHL than in cases of conductive or mixed hearing losses. Chandrasekhar et al. (2000) evaluated 50 patients and reported that of the 29% with hearing loss, 3% presented with sudden onset and 21% with gradual onset, with the remainder presenting with intermittent onset of the symptoms. Comparison of these studies suggests a greater tendency towards bilateral gradual onset of hearing loss rather than sudden onset (Khoza-Shangase, 2010).

Numerous clinical and medically oriented studies have exhibited the occurrence of hearing loss and other auditory manifestations in HIV/AIDS. According to the literature, auditory abnormalities associated with HIV/AIDS and its treatments have been reported in persons with varying degrees of HIV infection, among both symptomatic and asymptomatic patients, as well as in patients on ART (Khoza-Shangase, 2010). Indications exist which indicate that the HIV-effects on the auditory system can be direct as well as indirect (Larsen, 1998); however, this distinction is not always clear and consistent (Khoza-Shangase, 2010). Early reports in the literature showed that HIV infection might directly affect the auditory function due to the fact that the virus is neurotropic and commonly manifests itself neurologically (McArthur, 1987), which may be what Kallail, Downs and Schertz (2008) refer to as HIV/AIDS being the primary cause of auditory system disorders. Reportedly, these direct causes possibly give rise to central auditory pathology found in this population (Bankaitis; 1996; Lalwani and Sooy, 1992). More commonly, though, reports in the literature have focused on the indirect effects of the virus on the ear. It is believed that indirect causes that result in hearing loss arise from opportunistic infections, which require suppressive therapy, thereby leading to ototoxicity (Lalwani and Sooy, 1992; Bankaitis; 1996; Bankaitis and Schountz, 1998), and which Kallail et al. (2008) refer to as iatrogenic sources (Khoza-Shangase, 2010).

Larsen's (1998) more generalised delineation of ‘direct’ versus ‘indirect’ effects of HIV/ AIDS can be applied to the causes of auditory manifestations. Based on this distinction, it would seem that, generally, the majority of auditory manifestations can be attributed to indirect effects in the form of opportunistic infections (e.g. SNHL due to cytomegalovirus, otosyphilis, meningitis, encephalitis, otitis media, and tuberculosis of the ear), and neoplasms, with some direct effects including central hearing loss due to primary CNS lymphomas (Friedmann and Arnold, 1993), necrosis of the vestibulocochlear nerve due to herpes simplex and herpes zoster, and SNHL due to cryptococcal (Kwartler, Linthicum, Jahn and Hawke, 1991) and aseptic meningitis, as well as neurosyphilis (Little, Gardner, Acker and Land, 1995) and cytomegalovirus (Meynard, El Amrani, Meyohas, Fligny, Gozlan, Rozenbaum, Roullet and Frottier, 1997). Lastly, the side effects of HIV/AIDS treatment can also be viewed as indirect effects (Stern, Lin and Lucente, 1990; Lalwani and Sooy, 1992; Friedmann and Arnold, 1993; Gold and Tami, 1998; Larsen, 1998; Chandrasekhar et al., 2000; Khoza-Shangase, 2010).

A review of the literature with regard to causes of hearing loss in cases of HIV/ AIDS confirms the substantial role that opportunistic infections play in the development of auditory manifestations. Khoza and Ross (2002) stated that opportunistic infections in the form of intracranial events (such as encephalitis and meningitis), and syphilis and herpes contributed to the causes of hearing loss. Moreover, auditory manifestations in this population could be due to the fact that serous otitis media and associated conductive hearing loss have been found to be more common, presenting as one of the opportunistic infections (Gold and Tami, 1998; Khoza-Shangase, 2010).

Literature states that conductive hearing loss in this population is commonly caused by otitis media, which may be due to eustachian tube dysfunction (Gold and Tami, 1998). Various factors have been associated with this eustachian tube dysfunction and middle ear effusions including decreased cell-mediated immunity, recurrent viral infections, non-malignant lymphoid hyperplasia of the adenoids, nasopharyngeal tumours, sinusitis, and allergic autoimmune reaction to HIV (Stern, Lin, and Lucente, 1990; Lalwani and Sooy, 1992; Friedmann and Arnold, 1993; Gold and Tami, 1998; Khoza-Shangase, 2010). Chandrasekhar et al. (2000) claim that otitis media, which is reportedly exceptionally scarce in adults with ‘normal’ health, affects up to 23% of HIV-infected patients.

A large body of evidence exists in the literature regarding causes of SNHL in HIV/AIDS (Real et al., 1987; Smith and Canalis, 1989; Birchall et al., 1992; Friedmann and Arnold, 1993). Larsen (1998) asserts that the precise cause of SNHL in people infected with HIV infection is undetermined, a claim corroborated by Lalwani and Sooy (1992) who argue that in up to half of HIV-infected people with hearing loss, no cause can be found. Friedmann and Arnold (1993) associate SNHL in HIV/ AIDS to involvement of the central nervous system (CNS) and of the sensory-end organs, which may be due to neurosyphilis and neoplasms. According to these authors, these conditions may be complicated by opportunistic infections, such as cryptococcus and cytomegalovirus, as well as the side effects of some drugs used to treat the opportunistic infections (Khoza-Shangase, 2010).

Because of all the diseases and infections that the population with HIV/ AIDS presents with, it is not startling to find patients with hearing loss attributable to ototoxicity, as this population undergoes treatment that often involves potentially ototoxic medications (Birchall et al., 1992; Khoza-Shangase, 2010). Bankaitis and Schountz (1998) report that the use of experimental ARVs with undetermined side effects contributes to this occurrence of hearing loss. Additionally, ototoxic drugs that are often utilized in the management of opportunistic infections (such as tuberculosis) may raise the possibility for drug-induced hearing loss in this population.

The current study which aimed at determining the auditory manifestations of AIDS was therefore conducted in order to increase the evidence base that is more contextually relevant.

## Methodology

### Research Aim and Objectives

#### Primary Aim

To investigate the auditory status in a group of adults with AIDS prior to commencement of antiretroviral therapy in a hospital outpatient clinic in Gauteng, South Africa.

#### Specific Objectives

1. To estimate the prevalence of hearing loss and the presence of other otologic effects over and above hearing impairment (tinnitus, aural fullness, disequilibrium, and so forth)
2. To assess the type, degree and configuration of the hearing loss
3. To explore the type of hearing symptom onset (e.g. sudden or gradual/progressive onset)
4. To document case history data such as signs and symptoms of each participating participant and to identify any associations between obtained signs and symptoms and hearing loss

#### Design of the Study

As an extensive literature search yielded a paucity of both South African and internationally published data on this topic, the study was exploratory. The design utilized was prospective and qualitative in nature (Devore, 1999). The aim was to investigate the auditory status in a group of adults with AIDS prior to commencement of antiretroviral therapy; hence all measures were taken before commencement of ART.

#### Participants

A total sample of 150 participants comprised the study. The patients selected for this study were recruited from the Hospital's Adult HIV/AIDS clinic. Patients who attend this clinic have already been diagnosed with HIV/AIDS and are seen there for general medical management as well as antiretroviral treatment and monitoring. At the time of the study all patients with CD4+ counts below 200 cells/mm^3^ were assessed and counselled for ART at this clinic - and the current study targeted this group before they enrolled in the treatment.

#### Participant selection criteria

Due to the exploratory nature of the current study; and given the fact that little, if any, published research has been conducted on this aspect of HIV/AIDS in South Africa, the researcher believed that it was crucial to have a sample that was as closely representative of the general AIDS population as possible; while closely recording medical history that could have had an influence on the results. This medical history was obtained from both case history interviews and from medical records. To this end, criteria stipulated in Table 1 were observed.

**Table 1:** Summary of participant Inclusion Criteria and Recorded history

Criterion	Inclusion	History recorded
HIV/AIDS positive serology	**Yes**	√
History of ART		√
Age between 18 and 50 years	**Yes**	
Alert and oriented	**Yes**	
Noise exposure		√
Recent (less than 3 years) or current history of treatment for TB and radiotherapy		√
Positive clinical or serological evidence of syphilis		√
Middle ear pathology		√
Medical ear related history		√
Family history of hearing loss		√

#### Recruitment and Sampling Procedure

A nonprobability convenience sampling technique was utilized in the study since the sample was restricted to a part of the population that was readily available (Devore, 1999; Schiavetti and Metz, 2002), and true random sampling would have been difficult to achieve due to time, cost, and equipment limitations. Participants were approached at the clinic and asked to volunteer to participate in the study following an explanation of what the study entailed.

#### Research procedures and materials

Participants underwent case history interviews and medical record reviews, otoscopy and tympanometry, as well as conventional pure tone audiometry testing. Data were collected from assessing participants' dependant variables before administration of ART. Following infection control measures proposed by Kemp and Roeser (1998), all testing was conducted in a sound-proof booth.

- **Case History:**
  A case history form that targeted the signs and symptoms of auditory manifestations was utilized in order to gather all the important case history information, audiological data and some medical variables that could have exerted an impact on the results of the study.
- **Otoscopy:**
  The researcher evaluated the participants' ears for the presence of impacted wax; otitis externa; possible otitis media; perforated tympanic membranes; collapsed ear canals; presence of any growths and any other ear disorders (Friedman and Arnold, 1993). These otoscopic abnormalities are reported to have a significant effect on DPOAE and therefore needed to be documented before testing commenced (Hall, 2000).
- **Tympanometry:**
  Tympanometry (through the use of the Inter-Acoustic AZ26 audiotympanometer) was utilized to assess the status and integrity of middle ear functioning. Standard single frequency tympanometry using an 85dB SPL tone set at 226Hz was done.
- **Pure tone audiometry:**
  Conventional (250Hz–8000Hz) pure tone audiometry was performed on all participants through the use of the Inter-Acoustic AC 40 diagnostic audiometer. The criteria used to define normal hearing, was that of pure tone thresholds of 25dBHL or lower across all frequencies, with the absence of an air-bone gap (Martin and Clark, 2003). Where pure tone air conduction and tympanometry were abnormal at any test frequencies, bone conduction testing was conducted to determine the type of hearing loss. Participants presenting with abnormal findings were referred to an Ear, Nose and Throat Specialist for assessment and management, and were subsequently offered appropriate audiological rehabilitation.

#### Validity and Reliability

Test reliability was controlled and maintained at a high level by standardizing test administration, ensuring proper equipment calibration, and controlling patient variables. For all audiological assessments precautionary measures advocated by Bess and Humes (1990) and Hall (2000) were followed in terms of proper maintenance and calibration of the equipment; optimizing testing environment; correct earphone and bone vibrator placement, and proper probe placement for tympanometry. All testing was conducted in a soundproof booth or sound-treated room with equipment that was calibrated on an annual basis, with biologic calibration conducted before every test session. All participants were tested by the same researcher using the same test procedure. Furthermore, all patients were tested in the mornings to reduce the effect that fatigue can have on patients' responses to behavioural audiometry testing.

#### Data Analysis and Statistical Procedures

Participants' results were descriptively analysed and classified as either normal or abnormal. Normal hearing was taken as normal tympanometric results in the presence of normal otoscopic findings with pure tone responses at or better than 25 dB HL, with hearing loss being thresholds greater than 25dB HL with air-bone gaps greater than 10dB (Bess and Humes, 1990; Silman and Silverman, 1991). This stage of analysis aimed at determining the prevalence of hearing loss in the sample evaluated. For all participants presenting with abnormal auditory function, findings were further categorized into type of hearing loss; severity or degree of the loss; and type of onset of the hearing loss.

The hearing losses were classified into the three well-documented types of hearing losses (conductive - CHL; mixed - MHL; and sensorineural - SNHL) (Bess and Humes, 1990; Jacobson and Northern, 1990). SNHL was not further differentiated into cochlear (sensory) versus retrocochlear (neural), and this is acknowledged as another limitation of the current study.

The degree of hearing loss was determined using Silman and Silverman's (1991) classification of Magnitude of Hearing Impairment. This classification system, in line with Bess and Humes (1990), Gilbert, Smith and Stayner (2003), and Katz (1994) proposes that impaired hearing function begins at an average hearing level of 25 dB HL, and is categorized as seen in Table 2.

**Table 2:** System of classification of hearing loss in terms of degree of loss (Silman and Silverman, 1991) used in the current study

Average Hearing Level dB	Description
< 26 dB	Normal range
26dB – 40 dB	Mild hearing loss
41dB – 55 dB	Moderate hearing loss
56dB – 70 dB	Moderately severe hearing loss
71dB – 90 dB	Severe hearing loss
>91 dB	Profound hearing loss

Furthermore, in classifying the degree of the hearing loss, the researcher looked at the configuration of the loss and added categories depicting this change in degree of loss at frequency ranges (i.e. mild-moderate; severe-profound).

Configuration of hearing loss was established by describing the pure tone results as depicting flat, irregular, rising (low frequency), sloping (high frequency) configuration (Katz, 2002). Symmetry of hearing loss was also examined where the researcher established whether the hearing loss was unilateral or bilateral, and whether it was symmetrical or asymmetrical (Katz, 2002). The type of onset of hearing symptoms was also analysed where descriptors such as gradual, gradual/progressive, sudden, and sudden/progressive onset were used to define the manner in which the symptoms presented, based on patient reports. All significant case history factors and Ear, Nose and Throat Specialists' reports were recorded and analyzed along with the audiological results in order to obtain a comprehensive assessment and to ensure that participants presenting with hearing loss deemed (based on Ear, Nose and Throat Specialists' reports) to be opportunistic infection related were identified.

#### Ethical Consideration

Prior to commencement of the study, permission to conduct the research project was sought from the University of the Witwatersrand Human Research Ethics Committee (Medical) which gave unconditional ethical clearance in the form of protocol number M041131. The researcher ensured that permission to conduct the study was obtained from the Hospital management and from the Heads of the Audiology and HIV/AIDS clinics at the research site. Written informed consent to participate in the study was obtained from all participants before the study was conducted with an assurance that confidentiality of all records would be maintained. Furthermore, to ensure anonymity, the researcher ensured that no personal or identifying information was included in the research report and research coding numbers instead of identifying information were used. The current study also reduced risks to the participants to a minimum by conforming to the ethical principles (South African Medical Research Council, 2003) and observing provisions of the Nuremberg Code of ethics (Newell and Burnard, 2006) during the study. Lastly, the hospital and participants were given the opportunity to request to see the research results if they were interested.

## Results and Discussion

### Description of Participants

As depicted in Table 3, the sample included 53 (35%) males and 97 (65%) females between the ages of 20 and 46 years with a mean age of 33.9 years. The average CD4+ count was 124 cells/mm^3^ which was consistent with the CD4+ requirement that persons need to have before they can be enrolled in an ART programme in South Africa (i.e. CD4+ has to be below 200 cells/mm^3^).

**Table 3:** Demographic and CD4 count data of participants (N = 150)

FACTOR	SUB-CATEGORY	NO.
Age (Years)	Range Mean	20–46yrs 33.9yrs
Gender	Male Female	53 (35%) 97 (65%)
Ethnic Group	Black White Coloured Indian	141 (94%) 0 9 (6%) 0
CD4+ Count (cells/mm^3^)	Mean Standard deviation	123.5133 9.00294

Close inspection of the demographic profile of the participants in the current study reveals similarities between the sample evaluated and the general South African population infected with HIV/AIDS with regard to age, gender and race (Dorrington et al., 2006). This therefore implies a strong similarity to the South African population infected with HIV/AIDS, suggesting that the current study was performed on a sample that was fairly representative of the South African situation, particularly persons attending public health clinics. Dorrington et al. (2006) assert that women continue to have the highest HIV prevalence rates in the country. Most recent antenatal clinic data show that the prevalence rates amongst women exceeds 30% and that of men just over 25% (Dorrington et al., 2006). As in the rest of sub-Saharan Africa, HIV has been noted to disproportionately affect more women than men (Shisana and Simbayi, 2005). This gender bias in the prevalence rates of the disease was also evident in the sample recruited for the present study, with a much higher number of participants in the study being female.

### The prevalence rate of hearing loss

**Figure 1:**
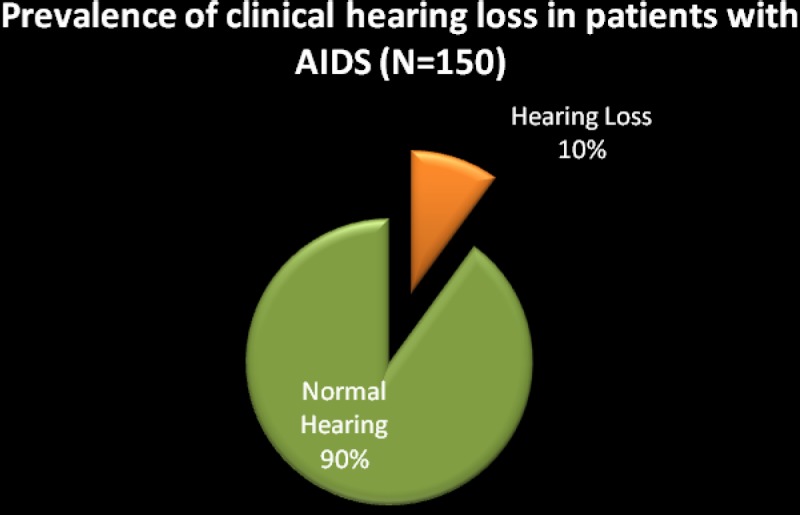
The prevalence rate of hearing loss in the sample of patients with AIDS (N = 150)

**Figure 2:**
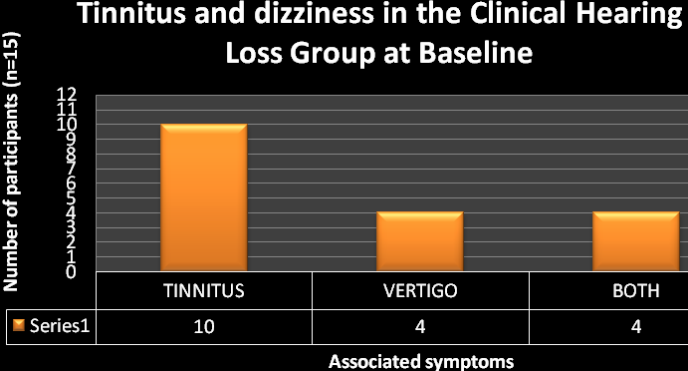
The occurrence of tinnitus and dizziness in the group of participants with clinical hearing loss (n=15)

Of the total sample of 150 participants evaluated, 135 (90%) had normal hearing, and 15 (10%) presented with a clinical hearing loss (Figure 1). Over and above changes in audiological function, participants with clinical hearing loss in the current study also presented with associated symptoms of hearing loss in the form of tinnitus and dizziness (Figure 2). Ten (67%) of the participants presented with associated tinnitus, 4 (27%) experienced dizziness, and 4 (27%) had simultaneous experience of dizziness and tinnitus. The significantly lower prevalence of dizziness in comparison to tinnitus is reassuring since this sign is believed to be more debilitating to the patient and can significantly affect the patients' quality of life (Katz, 2002).

These findings are consistent with previous reports on auditory manifestations of HIV/AIDS. The 10% that presented with hearing loss in the current sample is considered to be high since this sample only consisted of participants in the AIDS stage and excluded the earlier stages of the disease. This sample also did not include the effects of medications on hearing function since participants had not enrolled in ART at the time of data collection. This high occurrence of hearing loss in the sample assessed underscores the need for more research into this population to enhance generalizability of results so that vital decisions can be made regarding the anticipated burden of disease. Given the prevalence and disease burden of undetected hearing impairment and the availability of effective treatments, it is important for audiologists to engage early in the assessment and management of this population before quality of life is severely compromised.

## Assessment of the type, degree, configuration, and symmetry of the hearing loss

### Types of hearing loss

**Figure 3:**
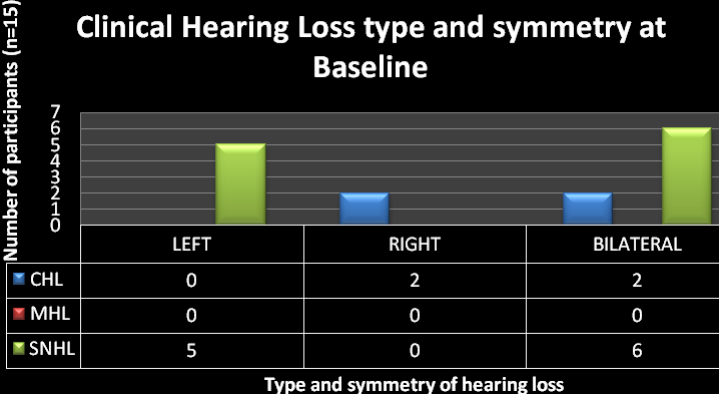
CHL = Conductive hearing loss; MHL = Mixed hearing loss; SNHL = Sensorineural hearing loss Types of hearing loss in the sub-sample of participants with clinical hearing loss (n =15)

Of the 15 participants with clinical hearing loss, 11 (73%) had sensorineural hearing loss (SNHL); 4 (27%) had conductive hearing loss (CHL); and none had mixed hearing loss (MHL) (Figure 3).

In reviewing the literature, the current findings provide support for the reports that claim the hearing loss seen in HIV/AIDS to be of any type (Chandrasekhar et al. 2000; Friedmann and Noffsinger, 1998; Khoza and Ross, 2002; Timon and Walsh, 1989). The limited occurrence of CHL was not a surprising finding particularly since this was consistent with previous findings (Khoza and Ross, 2002), of less occurrence of CHL in the AIDS stage even though otitis media has been reported to be most common in this population (Friedmann and Noffsinger, 1993; Gold and Tami, 1998; Lalwani and Sooy, 1992). The higher occurrence of SNHL in AIDS may be attributed to the progressive decline in patients' immunologic status which potentially places the patients at risk for being susceptible to the neurotrophic nature of the disease and to opportunistic infections, which have been found to cause hearing loss. Because of the permanent nature of SNHL when compared to CHL, findings from the current study highlight the need for increased awareness of this AIDS manifestation to ensure that rehabilitation in the form of diagnostic testing as well as amplification occurs. Moreover, counselling regarding communication enhancing strategies for both the patient and the family can be implemented early, thereby minimising the documented negative effects that a hearing impairment can have on an individual.

### Degree of hearing loss

**Figure 4:**
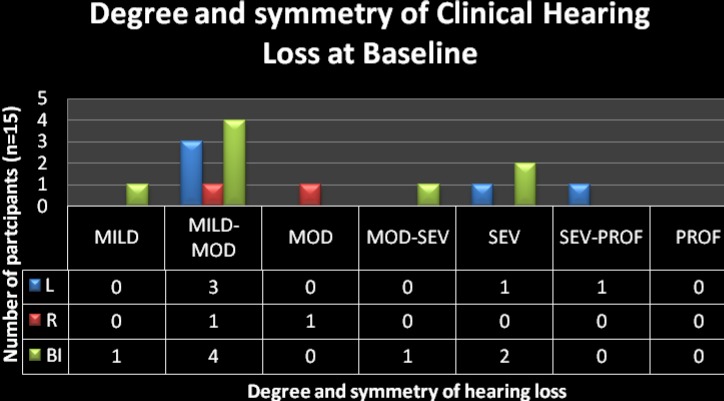
Key: Mod = Moderate; Sev = Severe; Prof = Profound; L = left; R = right; Bi = bilateral Degree and symmetry of hearing loss in the sub-sample of participants with clinical hearing loss (n = 15)

Following the classification of degree of hearing loss previously reported; it became evident that the hearing loss could occur in any degree of severity as depicted in Figure 4. However, the most prevalent degree of severity of hearing loss was the mild-moderate hearing loss followed by the severe degree of hearing impairment. Of the 15 participants, 9 (60%) presented with mild to moderate hearing loss while 3 (20%) presented with severe hearing loss.

These results are consistent with those reported in the literature where severity of hearing loss has been described as ranging from mild to severe with little mention of profound hearing loss - as was found in the current study. Despite the absence of the profound degree of hearing impairment in this sample, the implications for the patients' quality of life are still significant; and the role of the audiologist with regard to early and prompt effective services to this population is highlighted. Unaided severe hearing loss can have an influence not only on the patient's everyday listening environment; but in this case, may also have an influence on the patient's ability to follow treatment protocols; and subsequently adherence to ART.

### Configuration of hearing loss

No typical pattern of configuration of hearing loss could be established before antiretroviral treatment was instituted. In the total sub-sample (n = 15) of patients with hearing loss, 5 (33%) participants presented with a sloping/high frequency hearing loss while the remaining 67% presented with flat and/or irregular audiograms with an equal chance of involvement of all frequencies. These results are consistent with findings documented by Khoza and Ross (2002) on patients who were also not taking ART, where they found that all frequencies were affected equally or to varying degrees, depending on the possible cause of the hearing loss, and the hearing loss was not necessarily confined to high frequencies as previously reported. Several authors have reported a tendency towards sloping/high frequency hearing loss, and this finding was not the general trend observed in the current study. Findings from the current study were thought to vary due to the fact that the participants were treatment naïve, and so ototoxic hearing loss often presenting as high frequency hearing loss was not the main presenting manifestation.

### Symmetry of hearing loss

Of the total sub-sample of 15 participants with abnormal hearing, 7 (47%) had unilateral hearing impairment while 8 (53%) presented with bilateral hearing loss (Figure 3 and Table 3). Although there appears to be a dearth of reported data in respect to symmetry of hearing loss in adults infected with HIV/AIDS, the current study demonstrated that the hearing loss can be unilateral or bilateral, and can also be bilaterally symmetrical or asymmetrical (Table 3). This finding, when combined with the degree of hearing loss most prevalent in this population, stresses the importance of prompt audiological assessment and management of patients with AIDS. These findings highlight the crucial need for communication rehabilitation services by audiologists to minimise or eliminate the impact that the hearing impairment may have on the individual's communicative skills.

### Type of onset of hearing symptoms

Of the entire sample with hearing loss, all the participants (100%) presented with gradual/progressive hearing loss with no participant presenting with sudden onset of symptoms. These results are inconsistent with earlier reports by Khoza and Ross (2002) which revealed that sudden onset was mostly experienced by participants who presented with severe to profound SNHL, while gradual onset was mostly found in participants who presented with conductive and/or mixed hearing losses. In the current study, regardless of the type or degree of hearing loss, all participants presented with gradual onset of hearing loss. This type of onset of hearing loss probably explains the late patient presentation of symptoms to their physicians as patients may learn to compensate as the hearing loss gradually deteriorates.

### Case history information

Detailed analysis of the audiologic evaluation results together with the documented case history information where medical diagnoses had been confirmed by Ear, Nose and Throat Specialists (Tables 4 and 5) revealed the following:

- Patients who presented with SNHL had documented medical histories of meningitis; infections (syphilis and otosyphilis); and histories of ototoxic medication used in the treatment of TB and other opportunistic infections. Four participants had unknown causes of hearing loss.
- Patients who presented with CHL had a history of chronic suppurative otitis media, and otitis media with effusion.

**Table 4:** Case history and medical information for participants with clinical hearing loss (n=15)

Participant	Age (yrs)	Type of Hearing Loss	Medical History	Ear History
Participant 44s	30	Bilateral severe conductive hearing loss	Otitis media	Perforated tympanic membrane; Chronic suppurative otitis media
Participant 45s	41	L-mild-moderate sloping SNHL	Unknown	None
Participant 53	24	R-mild moderate CHL	Otitis media	Otitis media with effusion
Participant 54	32	Bilateral mild SNHL	? otosyphilis	None
Participant 61	46	L-Mild moderately severe SNHL	Unknown	History of otalgia
Participant 80	39	R-mild-moderate CHL	Otitis media	Perforated tympanic membrane; Chronic suppurative otitis media
Participant 83	33	Bilateral moderate severe SNHL	Otosyphilis	None
Participant 88	46	Bilateral moderate severe SNHL	Meningitis	None
Participant 89	41	L-moderate severe SNHL	Unknown	Hearing loss
Participant 97	29	Bilateral mild moderate sloping SNHL	TB treatment and syphilis treatment	None
Participant 102	20	L-severe profound SNHL	Syphilis & Viral meningitis	Hearing loss
Participant 104	39	Bilateral mild moderate SNHL	Unknown	None
Participant 2c	29	Bilateral sloping mild moderate SNHL	Herpes	None
Participant 44c	30	Bilateral severe CHL	Otitis media	Perforated tympanic membrane; Chronic suppurative otitis media
Participant 45c	41	L- mild moderate sloping SNHL	TB treatment and noise exposure	None

Key: L = left; R = right

**Table 5:** Summary of case history data and results for participants with clinical hearing loss (n=15)

FACTOR	SUB-CATEGORY	NO.	PERCENTAGE
Gender	Female Male	8 7	53 47
Age CD4+	Average Age Average CD4+	33.9yrs (Range 20–46yrs) 123.5 (Range 2–265 cells/mm^3^)	Not applicable
Hearing Function	Hearing loss	15	10
Type of Hearing Loss	Conductive Hearing Loss Sensorineural Hearing Loss Mixed Hearing Loss	4 11 0	27 73 0
Type of onset of Hearing Loss	Sudden Gradual	0 15	0 100
Symmetry of Hearing Loss	Unilateral Bilateral	7 8	47 53
Possible aetiology of Hearing Loss *	Meningitis Oto/syphilis Otitis Media Herpes TB Treatment Unknown Noise exposure	2 4 4 1 2 4 1	13% 27% 27% 7% 13% 27% 7%
Degree of Hearing Loss (n=15)	Mild Mild-moderate Moderate Moderate-severe Severe Severe-profound Profound	1 8 1 1 3 1 0	7 53 7 7 20 7 0
Tinnitus	Present Absent	10 5	67 33
Vertigo	Present Absent	4 11	27 73
Tinnitus & Vertigo	Present	4	27

* **% Scores do not add up to 100% as some participants presented with more than one possible aetiological factor.**

Possible causes of hearing loss found in the current study are consistent with literature reviewed. These findings were not surprising as these conditions are commonly seen in persons living with HIV/AIDS where immunological status has been severely compromised (Khoza and Ross, 2002; Larsen, 1998), and in up to 50% of HIV-infected people with hearing loss, no cause can be identified (Lalwani and Sooy, 1992). The fact that some of these causes are treatable causes which means reversible hearing loss once again highlights the importance of ensuring that patients with AIDS get audiologically monitored so that auditory manifestations can be identified early and Ear, Nose and Throat specialist management can be instituted to prevent the symptoms from becoming permanent.

## Conclusions

Limitations to the current study were present, and they included the fact that there was limited control over confounding variables such as previous exposure to ototoxic drugs. Furthermore, due to the sample size and the fact that the data were collected in one hospital in Gauteng, South Africa, the researcher's ability to generalize the results from the sample studied to the total population of adults with AIDS in South Africa is limited. From the findings of the current study it is clear, however, that there is great heterogeneity in presentation of the auditory manifestations of AIDS. With the exception of Khoza and Ross (2002), studies reviewed were mostly from developed countries where the presentation as well as the treatment of HIV/ AIDS may be different to that in developing countries. Hence, more investigation is required in this population, which underpins the rationale for intensified efforts into audiological research into HIV/ AIDS.

Audiological research into HIV/ AIDS may clearly demonstrate the potential role of the audiologist in both the assessment and treatment of patients AIDS. Most importantly, research conducted in specific contexts where the disease has varied effects as well as varied treatments may yield findings that are more relevant and evidence-based. Research conducted within the South African context; a context considered to be the epicentre of the African HIV pandemic; and a context that is acknowledged to present unique challenges of which political, social, economic, health, equipment, and personnel problems are deemed non-exhaustive; is crucial. Such research can directly inform the assessment and management protocols of this population, thereby impacting positively on their quality of life. These protocols can only be implemented if the audiological manifestations of AIDS and its treatments within a specific context have been clearly characterised.

Characterisation of the burden of disease has significant implications for developing countries where financial constraints guide the prioritisation of resources. For example, if the correct prevalence of audiologic manifestations of AIDS could be established, appropriate budget planning could be employed, which would have a direct influence on resource management for this population. Furthermore, when budgets are allocated for comprehensive HIV/ AIDS management, audiological services could be included in the programmes. Recommendations may need to be made for audiological services in terms of the provision of sensitive and objective equipment (such as measures of otoacoustic emissions [OAEs]) as well as the appointment of audiologists at HIV/AIDS clinics in state institutions.

While there are some clear auditory manifestations that were found in the current study, there is still a small evidence base. Findings regarding the prevalence as well as nature of auditory dysfunction in adults with HIV/ AIDS need to be replicated in other communities and populations in Africa, especially since sub-Saharan Africa has such high HIV incidence. Because of the vast variance in contexts, the differences in the patient populations studied, the wide range of methodological approaches employed, and so on — findings from international studies cannot easily be generalised to the African context. Furthermore, certain issues such as time of ART initiation, use of complimentary or alternative medicine in the African context, and how these may impact auditory function have not received attention. Hence, more context-relevant, current and updated research which utilises standardised and sensitive research instruments for ease of comparability of the findings is clearly needed to address this situation. Such research should also include more controlled studies to establish definitive links between HIV/ AIDS and its drug treatments with auditory function. Research should also include continuous reviews of prevalence and incidence data, as the nature of the disease and its manifestations may be changing. These research efforts would greatly enhance the development of a more consolidated body of evidence that can then be used to contribute to policy-making and programme design and implementation, while at the same time improving the efficacy of clinical practice.
